# Overexpression of the *FBA* and *TPI* genes promotes high production of HDMF in *Zygosaccharomyces rouxii*

**DOI:** 10.3389/fmicb.2024.1366021

**Published:** 2024-03-20

**Authors:** Yanhong Wang, Wei Liu, Jingyao Chen, Zhijiang Li, Yijia Hu, Zixiang Fan, Liangyuan Yan, Jiahui Liu, Yuao Zhou, Wei Jiang, Haiying Rui, Lingyan Dai

**Affiliations:** ^1^Heilongjiang Provincial Key Laboratory of Environmental Microbiology and Recycling of Argo-Waste in Cold Region, College of Life Science and Biotechnology, Heilongjiang Bayi Agricultural University, Daqing, China; ^2^Heilongjiang Agricultural Economy Vocational College, Mudanjiang, China; ^3^College of Food Science, Heilongjiang Bayi Agricultural University, Daqing, China; ^4^Daqing Branch of Heilongjiang Academy of Agricultural Sciences, Daqing, China

**Keywords:** 4-Hydroxy-2,5-dimethyl-3 (2H)-furanone (HDMF), *Zygosaccharomyces rouxii*, fructose-1,6-bisphosphate aldolase (FBA), triose phosphate isomerase (TPI), engineered yeast

## Abstract

4-Hydroxy-2,5-dimethyl-3 (2H)-furanone (HDMF) is widely used in the food industry as a spice and flavoring agent with high market demand. In this study, fructose-1,6-bisphosphate aldolase (FBA) and triose phosphate isomerase (TPI) were overexpressed in *Zygosaccharomyces rouxii* in the form of single and double genes, respectively, via electroporation. High-yield HDMF-engineered yeast strains were constructed by combining the analysis of gene expression levels obtained by real-time fluorescence quantitative PCR technology and HDMF production measured by HPLC. The results showed that there was a significant positive correlation between the production of HDMF and the expression levels of the *FBA* and *TPI* genes in yeast; the expression levels of the *FBA* and *TPI* genes were also positively correlated (*p* < 0.05). Compared with the wild type (WT), the engineered strains F10-D, T17-D, and TF15-A showed marked increases in HDMF production and *FBA* and *TPI* gene expression (*p* < 0.05) and exhibited great genetic stability with no obvious differences in biomass or colony morphology. In addition, the exogenous addition of d-fructose promoted the growth of *Z. rouxii*. Among the engineered strains, when fermented in YPD media supplemented with d-fructose for 5 days, TF15-A (overexpressing the *FBA* and *TPI* genes) generated the highest HDMF production of 13.39 mg/L, which is 1.91 times greater than that of the wild-type strain. The results above indicated that FBA and TPI, which are key enzymes involved in the process of HDMF biosynthesis by *Z. rouxii*, positively regulate the synthesis of HDMF at the transcriptional level. d-fructose can be used as a precursor for the biosynthesis of HDMF by engineered yeast in industrial production.

## 1 Introduction

4-Hydroxy-2,5-dimethyl-3 (2H)-furanone, commercially known as HDMF (Furaneol™), is a commonly available flavor compound in natural products with an aroma of brown sugar or baked goods (Feng et al., 2018). At low concentrations, HDMF smells like fresh strawberries and pineapple; hence, the name strawberry ketone or pineapple ketone exists (Negri et al., 2015). Because of its low threshold, low risk, and great effectiveness, HDMF is widely used in food, beverage, and daily chemical products as a spice and flavoring agent (Butter, 1999; Hu et al., 2020; Kocadağlı et al., 2020), with globally accepted recognition, which includes China GB 2760–2011, the American Flavor Manufacturing Association (FEMA No. 3174), and the Council of Europe (COE No. 536) (Luo, 2006). HDMF provides obvious aroma enhancement and modification with long-lasting effects even with the addition of trace amounts of HDMF. As a high-quality sweet flavor agent, HDMF has an aroma intensity 12 times and 4 times stronger than that of maltol or ethyl maltol, respectively (Pérez et al., 1999).

There are three main categories of HDMF synthesis: chemical synthesis, strawberry plant synthesis, and microbial synthesis (Pan et al., 2021; Li et al., 2023; Wang et al., 2023). Chemical synthesis methods are difficult to apply in industrial production because of the strict requirements for the production environment and operating conditions, high energy consumption, and high production costs, even if chemical synthesis is available. Products from chemical synthesis methods also potentially have shortcomings, such as poor aroma characteristics and solvent residues, making it impossible to replace natural HDMF (Zhou, 2006). Strawberry plant synthesis provides a natural source for HDMF; however, this method is limited due to its high cost and low yield, resulting in only a small amount of high-priced products with special requirements.

Microbial synthesis of natural spices has become a popular trend along with the rapid development and in-depth application of biotechnology, such as genetic engineering (Mindt et al., 2020). Research on the use of microbial fermentation to produce HDMF started in the 1980s, with slow progress mainly involving the use of *Lactobacillus*, *Pichia pastoris*, and *Z. rouxii* as microbial strains. *Z. rouxii* is the most commonly reported strain for use in the biosynthesis of HDMF and plays a critical role in the traditional fermented food flavoring industry. It has been used as a fermentation agent and flavoring strain in the brewing of soy sauce and bean paste, as well as an important yeast species in the production of fermented products such as Italian traditional balsamic vinegar, Japanese miso, and Thai fermented fish products (Han et al., 2020). As a representative salt-tolerant aromatic yeast, *Z. rouxii* has advantages in the formation of the aroma of fermented foods, which positively impacts the sensory and quality of the finished products (Dakal et al., 2014; Zhang et al., 2020). As a single-cell lower eukaryotic organism, yeast is safe, reliable, easy to cultivate, and has a controllable cost and simple operation because of its small genome, making it a popular host yeast for genetic engineering (Sehgal et al., 2018).

The biosynthesis of HDMF in strawberry fruits has been studied intensively, and although the complete route has not yet been fully characterized, substantial progress has been made. Fructose-1,6-diphosphate (FDP) was confirmed to be the most effective precursor by the isotope labeling method for HDMF production from strawberry fruits (Schwab and Schiefner, 2013). In 1996, Hecquet et al. reported that HDMF could be synthesized by *Z. rouxii* in media supplemented with 5% d-glucose and 10% FDP, and FDP was the most effective precursor among all the test samples (Hecquet et al., 1996). This conclusion laid the foundation for subsequent research. In 2001, Dahlen et al. confirmed through the isotope labeling method that the carbon on HDMF did not exchange with intracellular FDP but was directly derived from exogenously added FDP, further confirming that HDMF was a secondary metabolite formed by *Z. rouxii* using FDP (Dahlen et al., 2001). Other studies have shown that dihydroxyacetone phosphate (DHAP) is also an effective precursor for the synthesis of HDMF (Zabetakis et al., 1996; Cai et al., 2016). Both FDP and DHAP are intermediate products of the glycolysis (EMP) metabolic pathway. FDP is cleaved from six-carbon sugars to DHAP and 3-phosphoglyceraldehyde (GAP) by fructose-1,6-bisphosphate aldolase (FBA, EC: 4.1.2.13) in a reversible reaction (Say and Fuchs, 2010). DHAP and GAP are converted to each other via the action of triode phosphate isomerase (TPI, EC:5.3.1.1) (Xu et al., 1994). In our previous study, we completed cross-analyses and joint analyses and found that the two enzymes FBA and TPI play important roles in promoting the synthesis of HDMF in *Z. rouxii* in the presence of exogenously added d-fructose (Li et al., 2020). Therefore, it is expected that the accumulation of DHAP can be achieved by regulating the expression of *FBA* and *TPI* genes, thereby increasing HDMF production.

To study the effect of the enzymes FBA and TPI on the synthesis of HDMF by *Z. rouxii*, strains with *FBA* and *TPI* gene overexpression were constructed and screened for high yield of HDMF and genetic stability based on gene expression patterns and HDMF yields. The effect of the exogenous addition of d-fructose on HDMF production by the engineered strains was also studied. These results provide a theoretical basis for the regulation of the metabolic pathway of HDMF synthesis by *Z. rouxii* and lay the foundation for the industrial biosynthesis of HDMF using engineered strains of yeast.

## 2 Materials and methods

### 2.1 Construction of *FBA* and *TPI* gene overexpression vectors

*Z. rouxii* (freeze-dried powder, No. 32899) was purchased from the China General Microbiological Culture Collection Center. Primers were designed based on the *FBA* (ZYRO0E06556g) and *TPI* (ZYRO0G12408g) gene sequences. The *Z. rouxii* genome was used as a template to obtain amplified genes, and genomic DNA was extracted according to the method reported by Liu et al. (2022). The pECS-URA vector was purchased from Bio-Transduction Lab Co., Ltd. (Wuhan). The pECS-URA vector is a high-copy yeast expression vector with a full length of 6,631 bp, which can ensure the efficient expression of two genes in yeast at the same time. The *FBA* gene overexpression vector FBA-pECS-URA has a full length of 7,687 bp and was obtained by inserting the *FBA* gene into the multiple cloning site between 5′BamHI-SalI 3′ and the GAL1 promoter; the *FBA* gene was subsequently cloned from *Z. rouxii* and verified by sequencing. The *TPI* gene overexpression vector TPI-pECS-URA with a full length of 7,383 bp was generated in a similar way; only the cloning site was between the 5′NotI-NotI 3′ region, and the resulting promoter was GAL10. An *FBA-TPI* double gene overexpression vector was constructed in which the *FBA* gene and *TPI* gene were simultaneously inserted between the multiple cloning sites 5′BamHI-SalI 3′ and 5′NotI-NotI 3′, and the resulting construct was named TPI-FBA-pECS-URA, which has a full length of 8,439 bp. The detection primers and fluorescence quantitative PCR primers for *FBA* and *TPI* gene sequences were based on the promoter, terminator, ampicillin resistance gene, and other vector elements, as listed in Table 1.

**Table 1:** Primer sequences.

Name	Sequence (5′–3′)	Product length (bp)
FBA-F	GAAACTCATTTGCCCACCGCC	1,448
FBA-R	GGGTGAAAGATACGTGGGCGT	
TPI-F	CCTGCTAGCCACTGTTCCTCT	944
TPI-R	ACGTAGTCATCATCATTCACACGTA	
FG-F	TTGTGGAAATGTAAAGAGCCCCA	937
FG-R	TCAGCTTCCAACATACCATCGAA	
FC-F	GTTGGACTTGTCTGAAGAAACCG	750
FC-R	ACTCCTTCCTTTTCGGTTAGAGC	
AMP-F	CGTCGTTTGGTATGGCTTCATTC	581
AMP-R	TCCGCTCATGAGACAATAACCCT	

### 2.2 Yeast genetic transformation and screening of positive transformants

The FBA-pECS-URA, TPI-pECS-URA, and TPI-FBA-pECS-URA plasmids were transformed into *Z. rouxii* competent cells via electroporation. The competent cells were first treated with electroporation at a voltage of 2.1 kV for 5 ms, after which 1 mL of YPD medium was rapidly added to the electroporation cup. After gentle suspension, the cells were resuscitated at 28°C and 180 rpm for 2 h and then cultured for 2–3 days with the application of culture medium to solid YPD medium until a single colony was obtained. Once single clones were obtained, the yeast was treated with high temperature (ethyl acetate), and positive transformants were screened by colony PCR (Liu et al., 2022). These positive transformant colonies were picked and cultured in YPD liquid medium. After centrifugation, the yeast cells were vacuum freeze-dried and stored in a − 80°C freezer.

### 2.3 Yeast culture and biomass determination

Two types of yeast culture media were used in this study: YPD medium (containing 20.0 g/L peptone, 20.0 g/L glucose, 10.0 g/L yeast extract and 180 g/L NaCl) and YPD + Fru medium (120 g/L D-fructose was added to YPD medium). A small amount of lyophilized powder of *Z. rouxii* (including wild-type and positive transformants) was removed from an ultraclean workbench, added to 100 mL of YPD culture medium, and then left for fermentation at 28°C and 180 rpm for 3–4 days until the total number of cells reached approximately 2 × 10^8^ CFU/mL. Fermented yeast solution was inoculated into 100 mL of YPD culture medium at a 5% concentration for secondary culture at 28°C and 180 rpm for 30 ~ 35 h until the total number of cells reached 10^8^ CFU/mL. Then, the yeast fermentation broth was inoculated once again into 100 mL of YPD or YPD + Fru media at a 5% concentration for culture under the same conditions. Samples were taken at 0, 1, 3, 5, and 7 days of fermentation to measure gene expression and HDMF content, with three parallel samples in each group. The fermentation broth was mixed thoroughly, and 5 mL of the mixture was transferred to a centrifuge tube (dried to constant weight in advance) and centrifuged at 10,000 rpm for 10 min. After discarding the supernatant, the cells were washed three times with deionized water, placed in a 65°C oven, and dried to constant weight to weigh and calculate the biomass.

### 2.4 Determination of gene expression levels

Total RNA was extracted using the TRIzol method. After the RNA concentration and purity were detected with a nucleic acid protein analyzer, cDNA was synthesized using ReverTra Ace qPCR RT Master Mix with a gDNA Remover reverse transcription kit. The reaction conditions were as follows: 37°C for 15 min; 50°C for 5 min; 98°C for 5 min; and 4°C. SYBR^®^ Green Real-time PCR Master Mix was used for qRT-PCR with a 20 μL sample size under the following reaction conditions: 95°C predenaturation for 10 min, 95°C denaturation for 15 s, and 55°C annealing for 30 s for a total of 39 cycles. The FQ and TQ primers were used to analyze the *FBA* and *TPI* gene expression levels, respectively (Table 1). The *Z. rouxii GAPDH* gene was used as an internal reference (Liu et al., 2020), and the relative expression of the gene was calculated using the 2^−ΔΔCt^ method (Wohlfahrtova et al., 2015).

### 2.5 Determination of HDMF content

The HDMF content was determined via HPLC. The fermented yeast was transferred to a centrifuge tube and then centrifuged at 10,000 rpm for 8 min, after which the supernatant was passed through a 0.22 μm PES filter. The analytical column used was a ZORBAX Eclipse XDB-C18 column (5 μm, 250 × 4.6 mm), the mobile phase was 0.5% formic acid (A) and acetonitrile (B), the flow rate was 1.0 mL/min, the detection wavelength was 287 nm, the injection volume was 10 μL, and the gradient elution conditions were set according to the Zhou method (Zhou, 2018). The acetonitrile (B) was set as follows: 0 min, 5% B; 5 min, 10% B; 10 min, 20% B; and 20 min, 5% B.

### 2.6 Genetic stability analysis of engineered yeast

The yeast strains were continuously cultured on YPD liquid media for 20 generations. When the yeast cells reached the logarithmic growth phase, they were considered to have completed one generation, which was approximately 10–15 h in this study. The yeast fermentation broth of the previous generation was inoculated into YPD medium at a 5% concentration to continue cultivation and obtain the next generation. The genetic stability of engineered yeast strains from the 1st, 10th, and 20th generations was analyzed through genotyping validated by PCR, and HDMF production was measured by high-performance liquid chromatography (HPLC).

### 2.7 Data analysis

IBM SPSS Statistics 20.0 software was used to perform statistical analysis of the data using an independent-samples *t*-test to compare two groups through the least significant difference (LSD) test. *p* < 0.05, *p* < 0.01, or *p* < 0.001 indicated that the difference was statistically significant. WPS Excel 2016 was used for the data calculations, and Prism 8 was used for plotting.

## 3 Results and discussion

### 3.1 Cloning of the *FBA* and *TPI* genes

**Figure 1:**
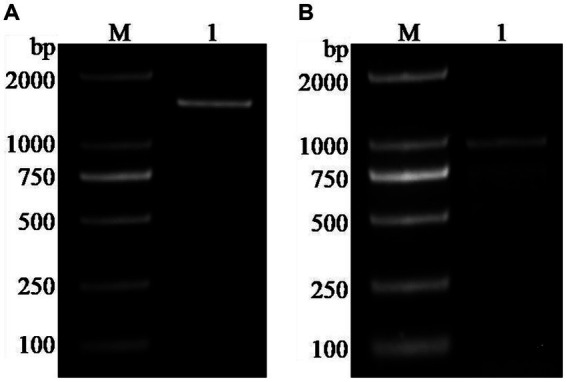
Cloning of *FBA* and *TPI* genes. **(A)** *FBA* gene; **(B)** *TPI* gene; M: marker (DL2000).

The *FBA* and *TPI* genes were cloned using the *Z. rouxii* genome as a template, and their target fragment sizes were 1,448 bp and 944 bp, respectively (Figure 1). The *FBA* gene is located on the E chromosome of *Z. rouxii*, with a cDNA length of 1,086 bp and encoding 361 amino acids. The *TPI* gene is located on the G chromosome, with a cDNA length of 747 bp and a length of 248 amino acids. Sequence homology comparison analysis indicated that the *FBA* gene had the highest similarity to *Candida glabrata* (93%), and the *TPI* gene had the highest similarity to *Saccharomyces kudriavzevii* (99%).

### 3.2 Construction of the gene overexpression vector

**Figure 2:**
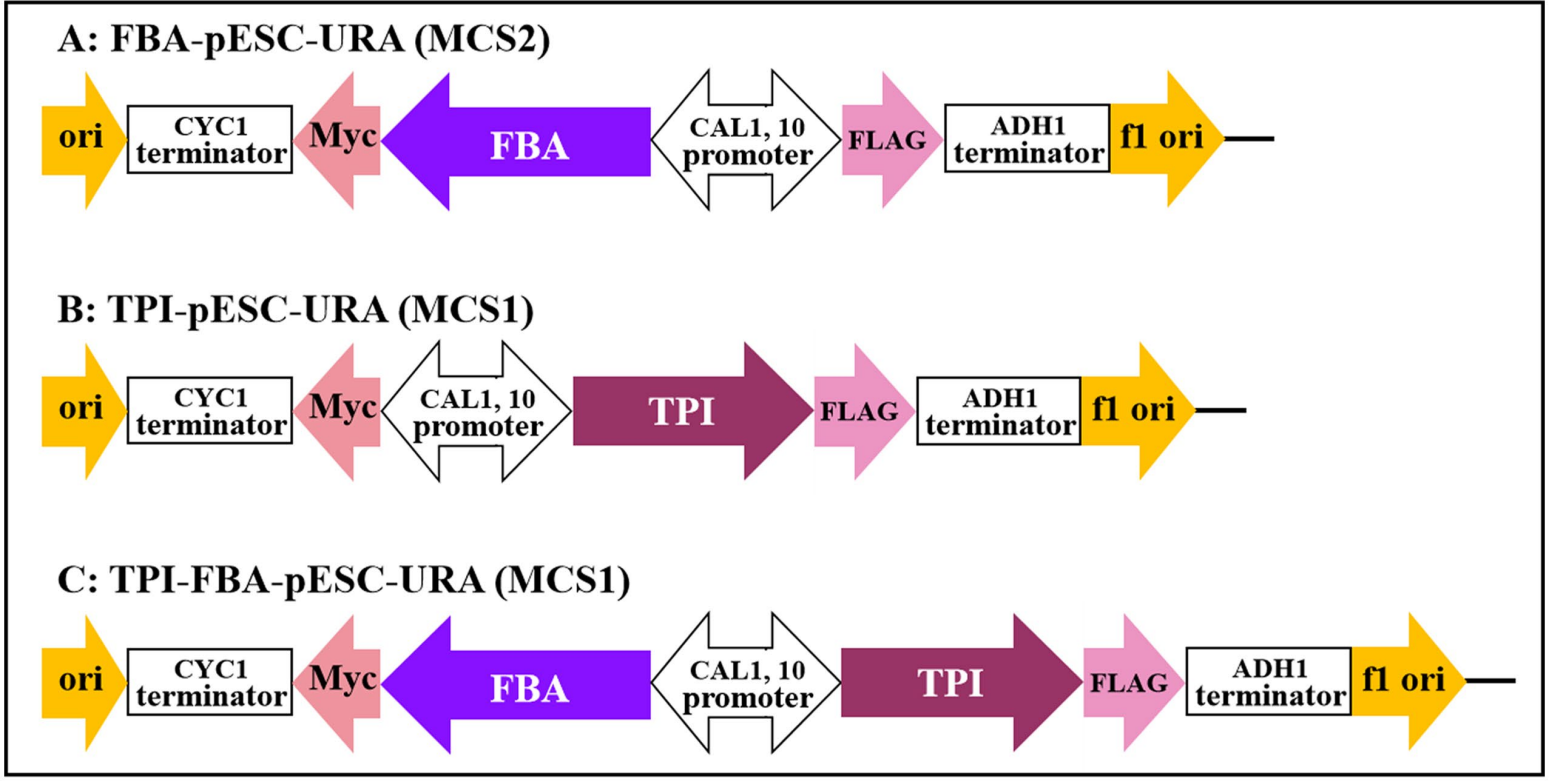
Construction of gene overexpression vector. **(A)** *FBA* gene overexpression vector; **(B)** *TPI* gene overexpression vector; **(C)** *TPI-FBA* double gene overexpression vector.

**Figure 3:**
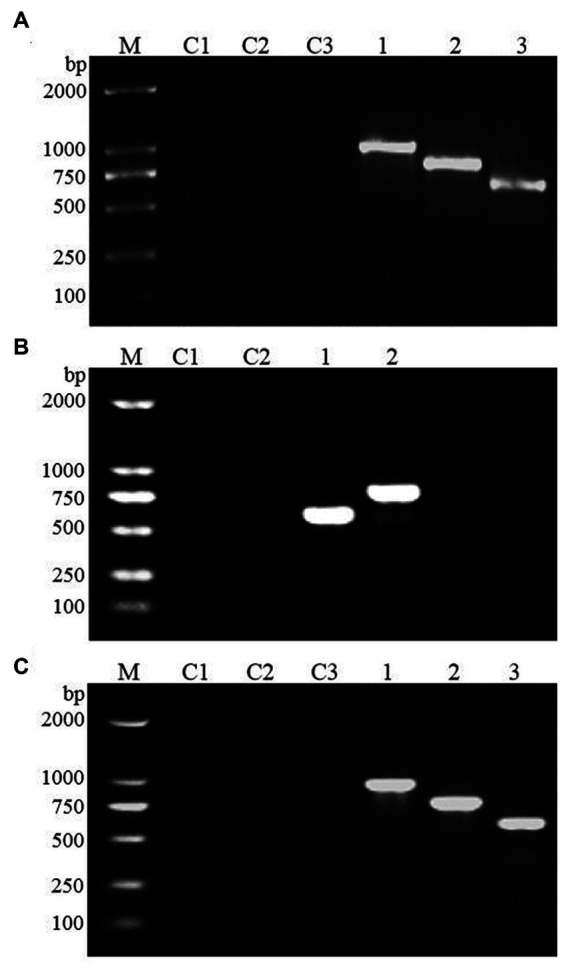
Electrophoretic map of vector validation by PCR. **(A)** FBA-pECS-URA; **(B)** TPI-pECS-URA; **(C)** TPI-FBA-pECS-URA; M: marker (DL2000); C1–C3: blank control (using deionized water as template); 1–3: PCR amplification products with different primers.

The *FBA* and *TPI* genes were connected to the GAL1 and GAL10 promoters of the high-copy yeast expression vector pESC-URA to construct the *FBA* gene, *TPI* gene, and *TPI-FBA* double gene overexpression vectors FBA-pECS-URA and TPI-pECS-URA and TPI-FBA-pECS-URA, respectively (Figure 2). To verify the FBA-pECS-URA and TPI-FBA-pECS-URA vectors, three pairs of primers, namely, FG, FC, and AMP, were selected to amplify the target band, while two pairs of primers, namely, TG and AMP, were used to verify the TPI-pECS-URA vector (Table 1). As displayed in the electrophoretic map of vector validation by PCR (Figure 3), all three vectors amplified specific target bands, and with sequencing resulting in the target fragments on the vectors, it was proven that all vectors were successfully constructed.

### 3.3 Screening of positive transformants

**Figure 4:**
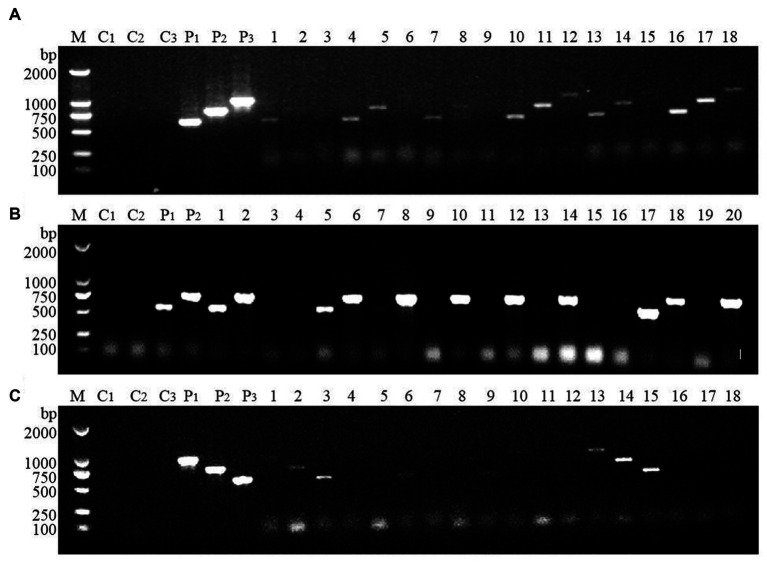
Screening of positive transformants. **(A)** *FBA* gene; **(B)** *TPI* gene; **(C)** *FBA-TPI* double gene. M: marker (DL2000); C1–C3: blank control (using deionized water as template, products amplified with different primers); P1–P3: positive control (using the corresponding plasmid as template, products amplified with different primers); 1–20: randomly selected single clone for *Z. rouxii*; in Figures **(A,C)**, the three consecutive lanes are the same colony, that is, 1–3 are the products amplified by the same colony using three pairs of different primers; in **(B)**, the two consecutive lanes are the same colony, that is, 1–2 are the products amplified by the same colony using two pairs of different primers.

**Figure 5:**
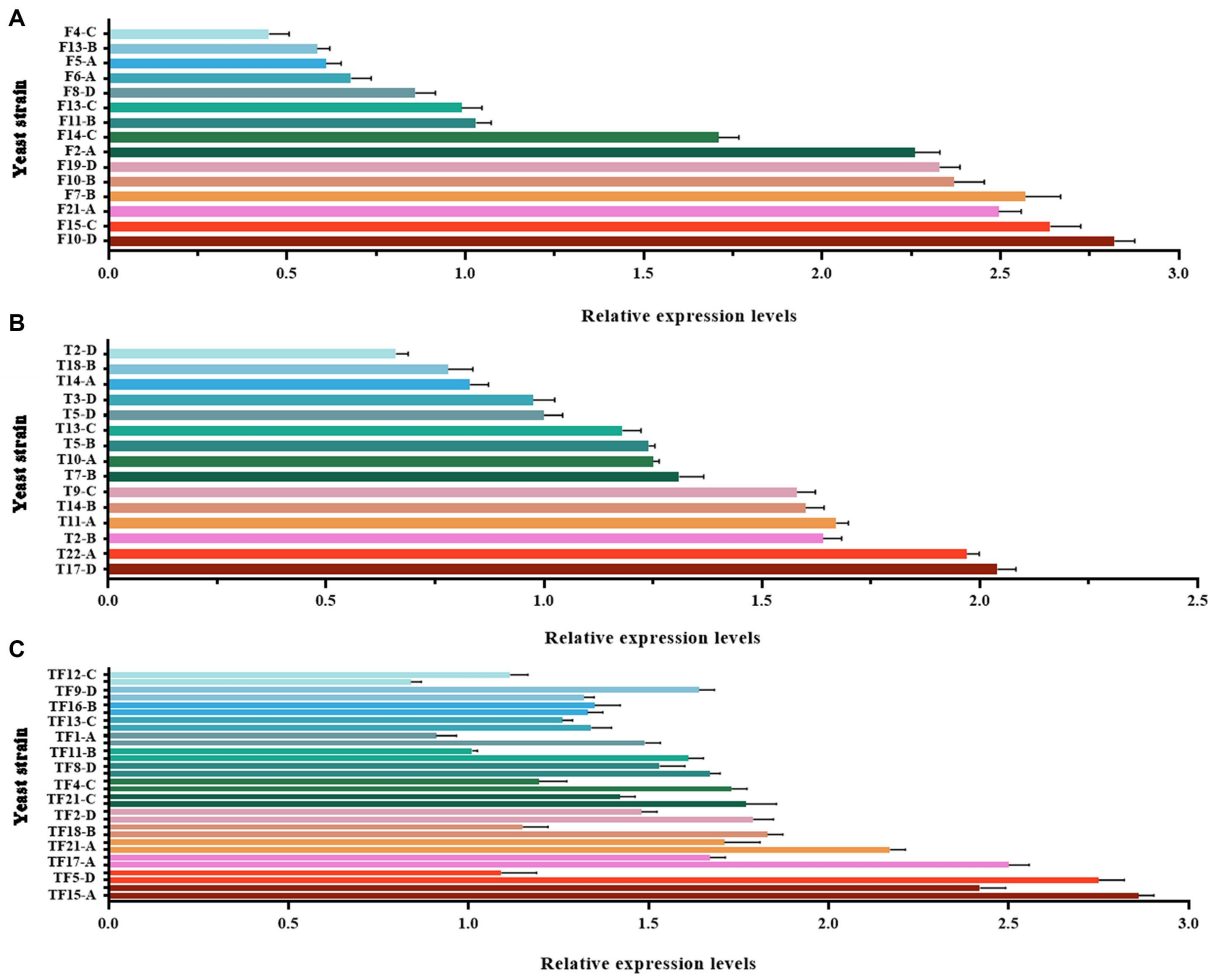
Relative expression levels of *FBA* and *TPI* genes in transformed yeast. **(A)**: *FBA* gene expression level in *FBA* gene overexpression transformants; **(B)**: *TPI* gene expression level in *TPI* gene overexpression transformants; **(C)**: *FBA* and *TPI* gene expression levels in *TPI-FBA* double gene overexpression transformants; the upper column is the relative expression of the *FBA* gene, and the lower column is the relative expression of the *TPI* gene. The *Z. rouxii GAPDH* gene was used as an internal reference, and the relative expression of the gene was calculated using the 2^−ΔΔCt^ method. The relative expression levels of transformed yeast strains were calculated using the expression level at each time point in WT.

Large-scale screening of positive transformants was conducted by colony PCR after the overexpression vectors were transformed into *Z. rouxii* by electroporation and single-clone colonies were generated via culture. Because *Z. rouxii* is the main yeast that produces the aromatic substance HDMF, there are currently no commercially available nutrient-deficient yeast strains available for theoretical research. Therefore, it is not possible to use URA3 screening markers on the pESC-URA vector for positive transformant screening. In this study, only multiple pairs of screening primers designed for gene overexpression vectors were used for large-scale colony PCR screening. The selection of the screening primer was the same as that used for the previous vector verification process, in which three pairs of primers were used to screen the *FBA* gene and *TPI-FBA* double gene overexpression transformants, while two pairs of primers were used to screen the *TPI* gene overexpression transformants. Some screening results are shown in Figure 4. Some colonies did not amplify the target band, some colonies amplified only part of the target band, and some colonies had all the target bands amplified; the latter were initially considered to represent positive transformations. Single colonies of *FBA*-, *TPI*-, and *FBA-TPI*-positive transformants were transferred to YPD liquid culture media for 3 days. Yeast cells were collected after culture, and gene expression was tested by qRT-PCR for further verification of positive transformants. The control group consisted of wild-type *Z. rouxii* cultured under the same conditions, and the internal reference gene used was *GAPDH*. The relative expression levels of each gene in the transformed yeast are displayed in Figure 5. Strains with high *FBA* gene expression included F10-D, F15-C, F21-A, F7-B, F10-B, F19-D, F2-A, and F14-C (Figure 5A); strains with high *TPI* gene expression included T17-D, T22-A, T2-B, T11-A, T14-B, T9-C, T7-B, T10-A, T5-B, and T13-C (Figure 5B); and strains with high expression levels of both *FBA-TPI* genes included TF15-A, TF5-D, TF17-A, TF21-A, TF18-B, TF2-D, TF21-C, TF4-C, TF8-D, and TF13-C, TF16-B, and TF9-D, etc. (Figure 5C).

### 3.4 Screening of high-yield HDMF yeast strains

The construction of engineered yeast strains with specific characteristics to obtain target products that integrate knowledge of fermentation engineering and genetic engineering has become a new trend in current research development (Rahmat and Kang, 2020). This approach can accumulate large amounts of product in a short time in an efficient and environmentally friendly manner. Zhou et al. (2021) increased campesterol production to 916.88 mg/L by modifying *Saccharomyces cerevisiae*. Zhang et al. modified the metabolic pathway of *Saccharomyces cerevisiae* and successfully increased the production of p-coumaric acid to 593.04 mg/L (Zhang et al., 2020). *Z. rouxii* is the most widely studied strain for biosynthesizing HDMF. It is possible to increase HDMF production by regulating its metabolic pathway through modification of its gene expression.

**Figure 6:**
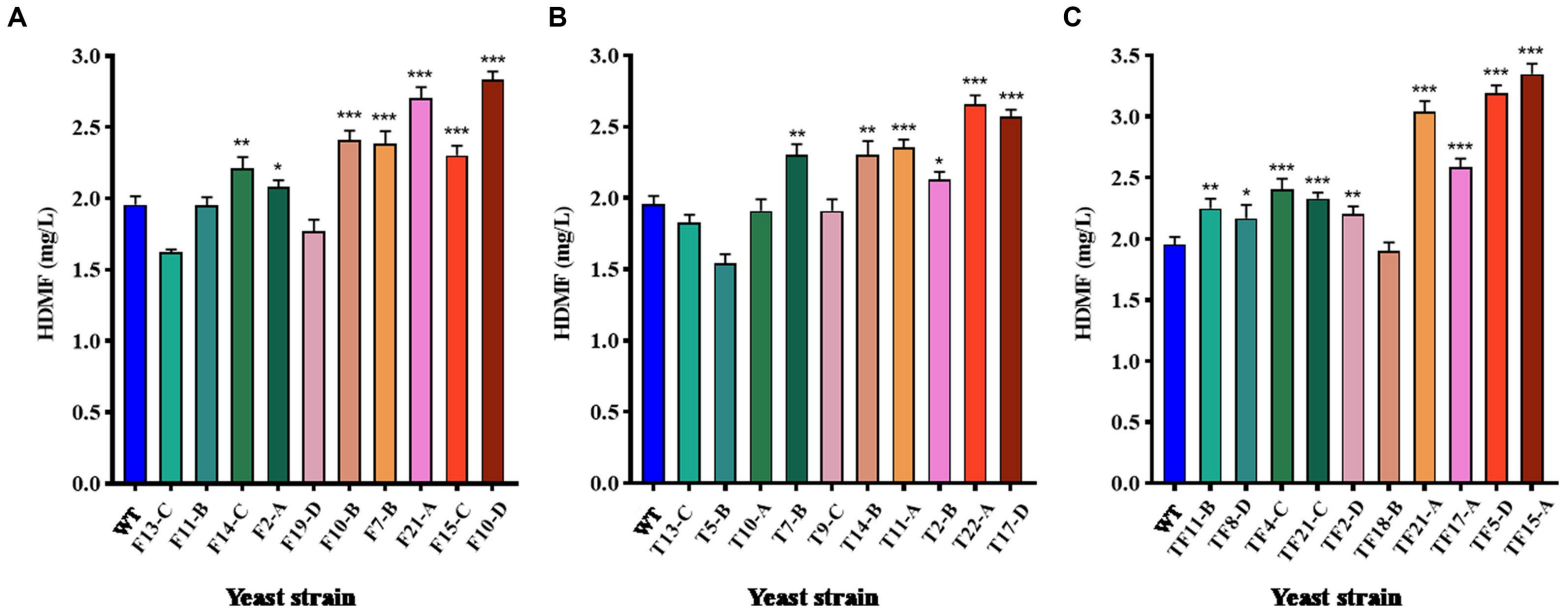
HDMF production by transformed yeast. **(A)** *FBA* gene overexpression transformants; **(B)** *TPI* gene overexpression transformants; **(C)** *TPI-FBA* double gene overexpression transformants; significance analysis is relative to the WT; * *p* < 0.05; ** *p* < 0.01; *** *p* < 0.001.

The top 10 strains with gene overexpression among the positive transformants were selected and cultured for 3 days. The HDMF yield was then measured using HPLC with the culture supernatant. WT was used as the control group, and the results are displayed in Figure 6. For *FBA* gene-overexpressing transformants, 70% of the strains had significantly increased HDMF production compared with that of the WT (*p* < 0.05), and strain F10-D had the highest yield of 2.83 mg/L (Figure 6A); 60% of the strains with *TPI* overexpression had significantly increased HDMF production (*p* < 0.05) compared with that of the WT, and the highest production of strain T22-A was 2.66 mg/L (Figure 6B); for *FBA-TPI* double gene-overexpressing transformants, 90% of the strains had significantly greater HDMF production (*p* < 0.05), and the highest production of strain TF15-A reached 3.35 mg/L (Figure 6C).

### 3.5 Correlation analysis between *FBA* and *TPI* gene expression levels and HDMF production

SPSS software was used to analyze the correlation between *FBA* and *TPI* gene expression and HDMF production. As the results listed in Table 2 indicate, the expression levels of the *FBA* gene and *TPI* gene were strongly positively correlated with HDMF production, and the correlation coefficients were 0.483 and 0.800, respectively (*p* < 0.01). This suggests that the high expression of the *FBA* and *TPI* genes positively regulates the synthesis of HDMF in *Z. rouxii*, possibly because these genes serve as key enzymes for the metabolic synthesis of HDMF and play an important role in the metabolic pathway of *Z. rouxii*. Moreover, the expression levels of the *FBA* gene and *TPI* gene were also significantly positively correlated, with a correlation coefficient of 0.515 (*p* < 0.05), indicating that the two genes influence each other in terms of gene expression.

**Table 2:** Correlation analysis between *FBA* and *TPI* expression levels and HDMF yield.

	*FBA* expression level	*TPI* expression level	HDMF yield
*FBA* expression level	1	0.515*	0.483**
*TPI* expression level	0.515*	1	0.800**
HDMF yield	0.483**	0.800**	1

**Figure 7:**
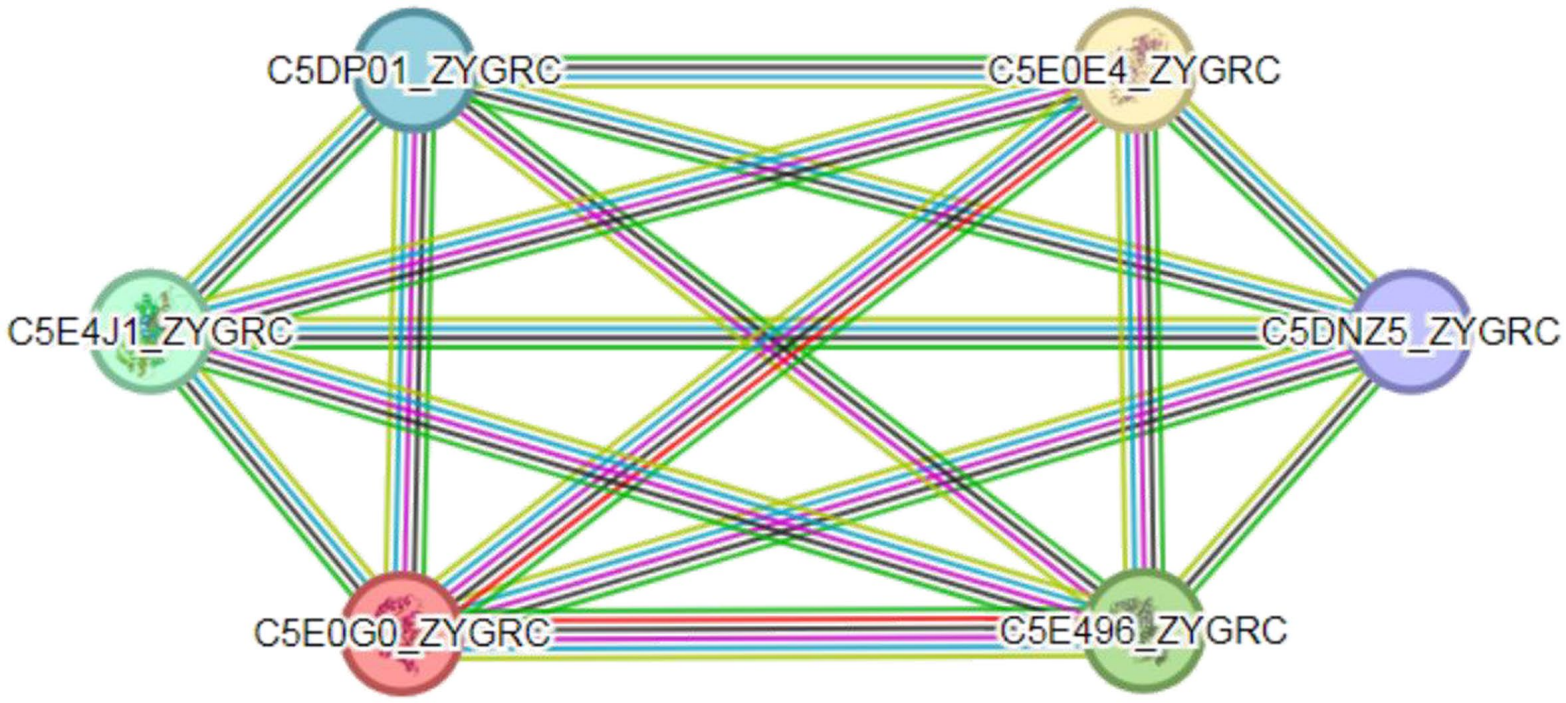
Protein interaction network between FBA and TPI. Colored circles represent proteins; empty nodes represent proteins of unknown 3D structure; and filled nodes represent a 3D structure that is known or predicted. Lines represent interactions between proteins; line color indicates the type of interaction evidence; and line thickness indicates the strength of the data support.

There are extensive interactions between proteins. Compared with single proteins, complexes formed by protein interactions are important components that perform major biochemical processes in cells (Macdonald et al., 2019). The prediction of protein–protein interactions is highly important for understanding the mechanism of action of organisms (Wu et al., 2016). The interactions between FBA and TPI proteins in *Z. rouxii* were analyzed through the STRING database and are illustrated in Figure 7. FBA protein (C5E4J1_ZYGRC) and TPI protein (C5E0GO_ZYGRC) interact in multiple pathways, including the glycolysis pathway, carbon metabolism pathway, pentose phosphate pathway, and fructose and mannitol metabolism pathways. The comprehensive score of FBA is 0.996, which is highly credible and yet to be verified since no supporting experimental data exist. In this study, a positive correlation between the expression of the *FBA* and *TPI* genes was confirmed, which provided evidence at the transcriptional level for possible interactions between the two genes. Additionally, the FBA and TPI proteins also interact with glucose-6-phosphate isomerase (C5DP01_ZYGRC), glyceraldehyde-3-phosphate dehydrogenase (C5E0E4_ZYGRC), phosphoglycerate kinase (C5E496_ZYGRC), and transaldolase (C5DNZ5_ZYGRC), and the comprehensive scores are all above 0.98.

### 3.6 Genetic stability analysis of transformed yeast

**Figure 8:**
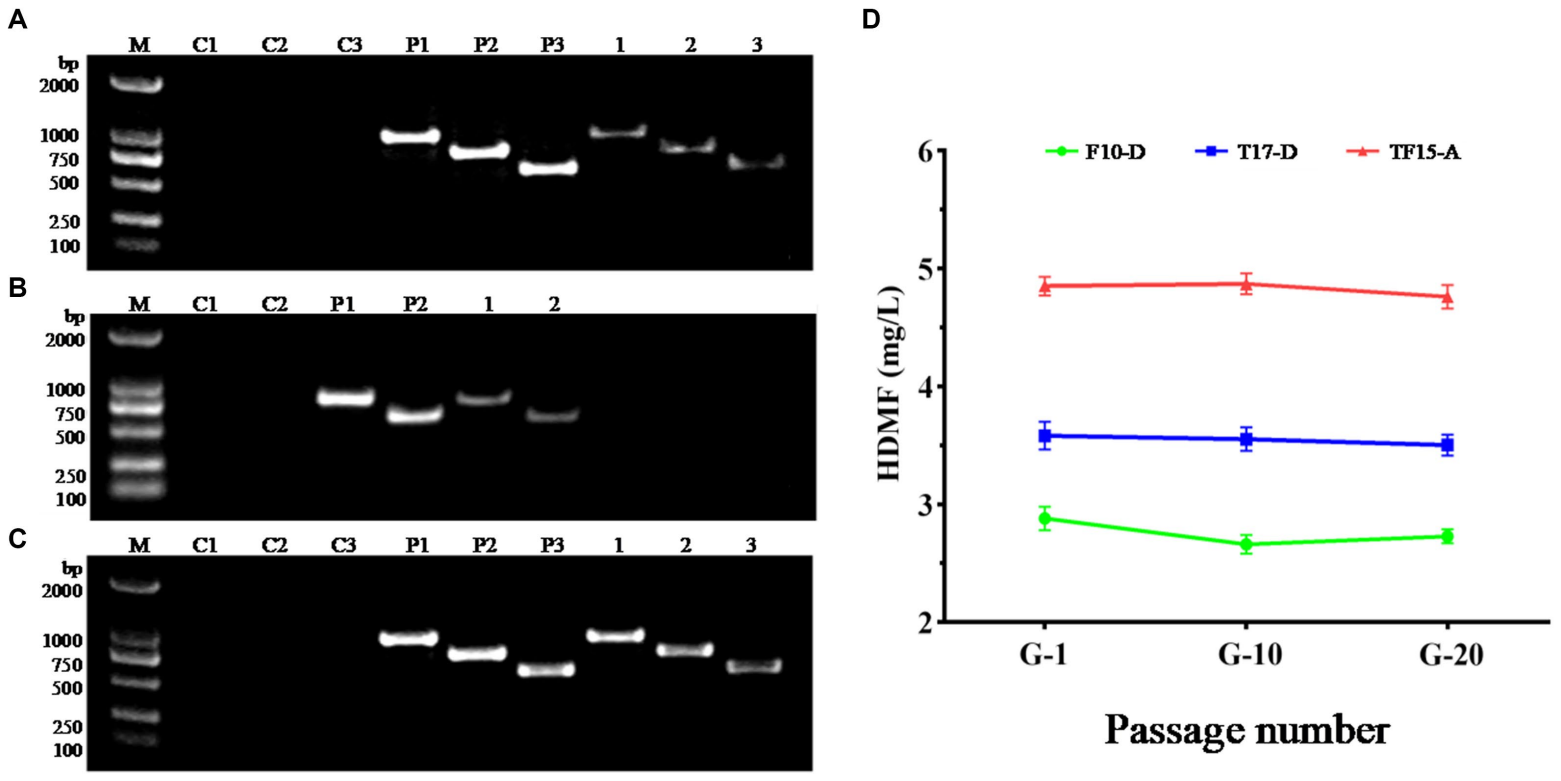
Genetic stability analysis of transformed yeast strains. **(A–C)** PCR electrophoretic map of the 20th generation of strains F10-D, T17-D, and TF15-A. **(D)** HDMF production of strains F10-D, T17-D, and TF15-A in the 1st, 10th, and 20th generation. C1–C3: Blank control (using deionized water as template); P1–P3: Positive control (using corresponding plasmid as template); 1–3: Using the 20th generation transformed yeast as template, products are amplified with different primers.

Due to the repair mechanism of the yeast strain itself, not all transformed strains can be genetically stable and maintain their excellent traits (Hülter et al., 2020). Therefore, the transformed strains F10-D, T17-D, and TF15-A were continuously subcultured, and the 1st, 10th, and 20th generations were selected for genetic stability testing. Single colonies of the 20th generation of the three strains were used as templates in the PCR amplification test to detect whether their genotypes had changed, and the results are shown in Figure 8. Specific target bands were all obtained, indicating that the genotypes of strains F10-D, T17-D, and TF15-A did not change after 20 generations (Figures 8A–C). At the same time, the HDMF production of three high-yield transformed strains was measured at the 1st, 10th, and 20th generations, and the results suggested that there was no significant change in HDMF production within 20 generations (Figure 8D). Collectively, these results confirmed the genetic stability of the F10-D, T17-D, and TF15-A strains.

### 3.7 Effects of the exogenous addition of d-fructose on the growth of transformed yeast and HDMF production

In 2003, Hauck et al. explored the optimal culture medium conditions for HDMF synthesis by *Z. rouxii* in yeast–protein–glucose media supplemented with FDP. Their results showed that the pH and NaCl concentration of the culture medium have a great impact on HDMF production and cell growth, the optimal pH is 5.1, and the optimal NaCl concentration is 20%. Additionally, when under high osmotic pressure, NaCl provides the best results (Hauck et al., 2003). However, FDP is expensive and is not suitable as a precursor for large-scale industrial production of HDMF. Luckily, d-fructose can generate FDP via the catalysis of two key enzymes in the EMP process, hexokinase (HK) and phosphofructokinase 1 (PFK1) (Schrøder et al., 2013; Hu et al., 2019; Stoddard et al., 2020). Our previous research revealed that HDMF production by *Z. rouxii* fermented in YPD medium supplemented with 120 g/L d-fructose and 180 g/L NaCl increased significantly at 3, 5, and 7 days (*p* < 0.05) (Li et al., 2020).

In this study, the biomass of transformed yeast, changes in HDMF production, and *FBA* and *TPI* gene expression levels were tracked and analyzed to explore the impact of the exogenous addition of d-fructose on *FBA*- and *TPI*-overexpressing transformed yeast. Based on the expression levels of the *FBA* and *TPI* genes in the transformed yeast and their correlation with HDMF production, F10-D, T17-D, and TF15-A were selected as high-yield HDMF strains for dynamic culture.

#### 3.7.1 Effect on biomass

**Figure 9:**
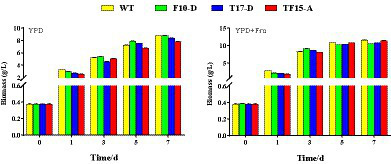
Biomass of transformed yeast in two different culture media. Yeast strains included wild-type *Z. rouxii* (WT) and three transformed yeast strains (F10-D, T17-D, and TF15-A strains). The yeast culture media used were YPD media and YPD + Fru media (120 g/L d-fructose was added to YPD media). Biomass of yeast was detected at 0, 1, 3, 5, and 7 days of fermentation.

The biomass of *Z. rouxii* WT and transformed yeast in YPD and YPD + Fruit media are shown in Figure 9. In both media, there was no obvious difference in the biomass of the WT and transformed yeast at each stage of the fermentation process. However, compared with that in the YPD group, the biomass of the WT and transformed yeast in the YPD + Fru group increased significantly at 1, 3, 5, and 7 days, indicating that d-fructose promoted the growth of *Z. rouxii*. Research has indicated that the exogenous addition of different carbon sources could impact the growth rate of yeast. Liu and Li confirmed that glycerol can promote the growth of *Candida tropicalis* by adding different carbon sources to the culture medium (Liu and Li, 2021); Song discovered that when *Glycerogenic candida* was cultured in media supplemented with polysaccharides and glucose as carbon sources, its biomass increased significantly (Song, 2012).

#### 3.7.2 Effect on *FBA* and *TPI* gene expression

**Figure 10:**
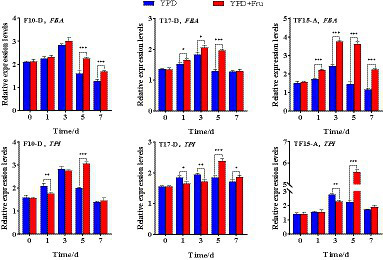
Relative expression levels of the *FBA* and *TPI* genes in transformed yeast in two different culture media. Yeast strains included wild-type *Z. rouxii* (WT) and three transformed yeast strains (F10-D, T17-D, and TF15-A strains). The yeast culture media used were YPD media and YPD + Fru media (120 g/L d-fructose and 180 g/L NaCl were added to YPD media). Gene expression levels were determined at 0, 1, 3, 5, and 7 days of fermentation. FQ and TQ primers were used to analyze *FBA* and *TPI* gene expression levels, respectively (Table 1). The *Z. rouxii* *GAPDH* gene was used as an internal reference, and the relative expression of the gene was calculated using the 2^−ΔΔCt^ method. The relative expression levels of transformed yeast strains were calculated using the expression level at each time point in the WT. Significance analysis was performed on two different culture media at each time point: *p < 0.05; **p < 0.01; ***p < 0.001.

The *FBA* and *TPI* gene expression levels of the F10-D, T17-D, and TF15-A strains in YPD and YPD + Fru media at different fermentation times are summarized in Figure 10. Compared with those in the YPD group, the *FBA* and *TPI* genes of the F10-D, T17-D, and TF15-A strains in the YPD + Fru group were upregulated markedly on day 5 (*p* < 0.001). The *FBA* gene of the three transformed yeast strains presented the highest expression level on day 3 in both media, and the *TPI* gene was expressed at the highest level on day 5 in the YPD + Fru media. On a relative basis, the *FBA* gene of the F10-D strain presented extremely significant upregulation on days 5 and 7 in YPD + Fru medium, with a 1.4-fold increase on day 5 (*p* < 0.001) compared to that in the YPD group; the *FBA* gene of the T17-D strain in YPD + Fru medium was upregulated on days 1, 3, and 5, with a 1.5-fold increase on day 5 (*p* < 0.05); the *FBA* gene of the TF15-A strain was extremely significantly upregulated on days 1, 3, 5, and 7, and the expression level was upregulated almost 2.5-fold (*p* < 0.001) on day 5. The *TPI* gene showed a similar trend in the YPD + Fru group compared to that in the YPD group. The *TPI* gene of the F10-D strain was significantly upregulated at 1 and 5 days; in the T17-D strain, the *TPI* gene was upregulated at 1, 3, 5, and 7 days; and in the TF15-A strain, the *TPI* gene was upregulated at 3 and 5 days, and the expression level increased nearly 2.5 times on day 5 (*p* < 0.05). Overall, in YPD + Fru medium, the expression levels of the *FBA* gene and *TPI* gene in the TF15-A strain were greater than those in the F10-D and T17-D strains on day 5, further confirming the positive correlation between the *FBA* and *TPI* genes.

#### 3.7.3 Effect on HDMF production

**Figure 11:**
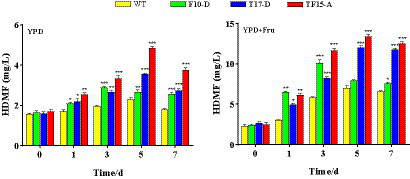
HDMF production by transformed yeast in two different culture media. Yeast strains included wild-type *Z. rouxii* (WT) and three transformed yeast strains (F10-D, T17-D, and TF15-A strains). The yeast culture media used were YPD media and YPD + Fru media (120 g/L d-fructose was added to YPD media). HDMF production was determined at 0, 1, 3, 5, and 7 days of fermentation. Significance analysis was carried out between transformed yeast strains and the WT at each time point, respectively. * *p* < 0.05; ** *p* < 0.01; *** *p* < 0.001.

The amount of HDMF produced by the three transformed yeasts in the two groups of culture media is shown in Figure 11. HDMF content increased to its peak and then decreased for all the groups, possibly because the gradual consumption of the substrate in the culture medium caused a decrease in HDMF production. HDMF production by the F10-D strain peaked on day 3 in all the culture media, while it peaked on day 5 for the T17-D and TF15-A strains. This finding is consistent with the gene expression trends for the *FBA* and *TPI* genes.

For the YPD medium group, the HDMF production of the three transformed strains significantly increased compared with that of the WT group and was significantly greater than that of the control group at 1, 3, 5, and 7 days (*p* < 0.05). Strain TF15-A reached a maximum yield of 4.86 mg/L after 5 days of fermentation, which was 2.11 times greater than that of the WT strain. The highest HDMF production of strains F10-D (fermented for 3 days) and T17-D (fermented for 5 days) was 1.47 and 1.54 times greater than that of the WT strain, respectively.

Compared with that in the WT group, the amount of HDMF produced by the three transformed strains in the YPD + Fru media group was also significantly increased (*p* < 0.05). TF15-A still provided the highest HDMF production, which was 13.39 mg/L (day 5) and was 1.91 times greater than that of the WT. The maximum HDMF production values of strains F10-D (fermented for 3 days) and T17-D (fermented for 5 days) were 1.73 and 1.71 times greater than those of the WT, respectively.

Compared with that in YPD media, the HDMF production of all test strains in YPD + Fru media increased significantly. Taking the transformed strain TF-15A as an example, its highest HDMF production value in YPD + Fru medium was 2.76 times greater than that in YPD medium. This may be because, with the addition of d-fructose, the flow of carbon metabolism changed in the EMP pathway (Li et al., 2020). At the same time, compared with that of YPD media, the osmotic pressure of YPD + Fru media was greater, and the *TPI* gene expression of the transformed strain was significantly greater, resulting in more metabolic flux being converted from GAP to DHAP (Xie and Zhang, 2011). An increase in the amount of precursor substances increased HDMF production. From the perspective of fermentation time, F10-D, which overexpresses the *FBA* gene, had the most HDMF production on the third day of fermentation, while T17-D, which overexpresses the *TPI* gene, and TF15-A, which overexpresses both *TPI-FBA* genes, had the highest HDMF yield on the fifth day of fermentation; this result reflects the sequence of enzymatic reactions in the EMP pathway. From the perspective of maximum HDMF yield, TF15-A reached a maximum of 13.39 mg/L, followed by T17-D at 12.03 mg/L. The minimum F10-D concentration was 10.11 mg/L, which indicates that the closer the metabolic process is to the enzyme (TPI) that produces HDMF, the greater the genetic engineering effect, and the greater the multienzyme linkage effect, with a positive correlation between upstream and downstream regions.

## 4 Conclusion

The *FBA* gene and *TPI* gene were cloned using the genome of *Z. rouxii* as a template, and the *FBA* gene, *TPI* gene, and *TPI-FBA* double gene overexpression vectors FBA-pECS-URA, TPI-pECS-URA, and TPI-FBA-pECS-URA were constructed, respectively. The correlation between HDMF production and *FBA* and *TPI* gene expression was analyzed after screening out three positive transformants of the *FBA* gene, *TPI* gene, and *FBA-TPI* gene overexpression. The results indicated that there was a significant positive correlation between HDMF production and the expression levels of the *FBA* and *TPI* genes in *Z. rouxii*, and the expression levels of the *FBA* and *TPI* genes were also significantly positively correlated (*p* < 0.05). The *FBA* gene-overexpressing strain F10-D, the *TPI* gene-overexpressing strain T17-D, and the *FBA-TPI* double gene-overexpressing strain TF15-A were screened out as high-yield engineered strains of HDMF. The HDMF production was remarkably greater than that of WT strains (*p* < 0.05), with no observable differences in biomass or colony morphology. Additionally, these high-HDMF strains had good genetic stability. With the exogenous addition of d-fructose promoting the growth of *Z. rouxii*, the most abundant strain, TF15-A, produced 13.39 mg/L HDMF when fermented in YPD + Fru medium for 5 days, which is 1.91 times greater than the amount produced by the WT strain.

## Data availability statement

The original contributions presented in the study are included in the article/supplementary material, further inquiries can be directed to the corresponding author.

## Author contributions

YW: Writing – review & editing, Writing – original draft, Software, Methodology, Investigation, Data curation, Conceptualization. WL: Writing – review & editing. JC: Writing – review & editing. ZL: Writing – review & editing, Writing – original draft, Software, Methodology, Investigation, Funding acquisition, Data curation, Conceptualization. YH: Writing – review & editing, Software, Data curation. ZF: Writing – review & editing, Software, Data curation. LY: Writing – review & editing, Software, Methodology. JL: Writing – review & editing, Software, Methodology. YZ: Writing – review & editing, Data curation. WJ: Writing – review & editing, Investigation. HR: Writing – review & editing, Software, Methodology. LD: Writing – review & editing, Supervision, Funding acquisition, Conceptualization.
